# Optimizing MFCC Parameters for Breathing Phase Detection

**DOI:** 10.3390/s25165002

**Published:** 2025-08-13

**Authors:** Assel K. Zhantleuova, Yerbulat K. Makashev, Nurzhan T. Duzbayev

**Affiliations:** 1 Department of Computer Engineering, International Information Technology University, Almaty 050040, Kazakhstan; 38565@iitu.edu.kz; 2 Institute of Genetics and Physiology, Almaty 050060, Kazakhstan

**Keywords:** respiratory phase detection, MFCC, optimization, SVM, deep learning

## Abstract

Breathing phase detection is fundamental for various clinical and digital health applications, yet standard Mel Frequency Cepstral Coefficients (MFCCs) settings often limit classification performance. This study systematically optimized MFCC parameters, specifically the number of coefficients, frame length, and hop length, using a proprietary dataset of respiratory sounds (*n* = 1500 segments). Classification performance was evaluated using Support Vector Machines (SVMs) and benchmarked against deep learning models (VGGish, YAMNet, MobileNetV2). Optimal parameters (30 MFCC coefficients, 800 ms frame length, 10 ms hop length) substantially enhanced accuracy (87.16%) compared to default settings (80.96%) and performed equivalently or better than deep learning methods. A trade-off analysis indicated that a clinically practical frame length of 200–300 ms balanced accuracy (85.08%) and latency effectively. The study concludes that optimized MFCC parameters significantly improve respiratory phase classification, providing efficient and interpretable solutions suitable for real-time clinical monitoring. Future research should focus on validating these parameters in broader clinical contexts and exploring multimodal and federated learning strategies.

## 1. Introduction

Breathing is a fundamental physiological process governed by complex neural circuits and homeostatic mechanisms, playing a central role in human health, cognition, and behavior [1,2,3,4]. Recent findings indicate that respiratory patterns, including phase transitions, are individually unique, stable over time, and predictive of cognitive and physiological states [5,6]. Automated detection and characterization of breathing phases—specifically, inhalation, exhalation, and pauses—are essential for objective assessment in respiratory medicine, digital therapeutics, stress management, and biofeedback applications [7,8,9,10,11]. The precise detection of breathing phases holds significant clinical relevance in medical diagnostics and health monitoring, enabling the identification of abnormalities associated with respiratory diseases such as asthma, obstructive sleep apnea (OSA) and chronic obstructive pulmonary disease (COPD) [12,13]. Recent statistics underscore this importance: OSA affects an estimated 38% of adults worldwide, while COPD and asthma exhibit a prevalence of approximately 10–11% [14,15,16]. Beyond diagnostics, monitoring breathing phases supports the control of physical load in sports, where respiratory patterns influence performance, focus, and recovery during exercise [17,18]. Furthermore, in psychophysiology, breathing patterns are intricately linked to emotional states, facilitating transitions in psychophysiological flexibility and contributing to mood regulation through practices such as structured respiration [19,20]. In this context, accurate and automated phase detection forms the basis for a range of clinical and digital health applications.

Traditional techniques, including spirometry, pneumography, plethysmography, capnography, accelerometry, and electromyography, despite their utility, present notable limitations, including invasiveness (e.g., requiring mouthpieces or electrodes), elevated costs for equipment and maintenance, and impracticality for long-term monitoring due to patient discomfort and restricted mobility [13]. To address these drawbacks, recent advancements have shifted toward non-invasive acoustic methods, which analyze sounds captured via microphones to identify breathing phases in real-time, offering greater accessibility and comfort for continuous health surveillance [21,22].

Mel Frequency Cepstral Coefficients (MFCCs) have long been recognized as the gold standard for perceptually relevant spectral analysis in speech and biomedical sound processing [23,24]. However, most studies employ default or empirical MFCC parameter settings, potentially limiting classification performance and model generalizability. Recent work has demonstrated that optimizing the selection of MFCC parameters, including the number of coefficients, frame length, and hop length, has been shown to be critical for improving the performance of respiratory disease classification systems [25].

While prior studies have primarily targeted disease detection using default settings, we focus on optimizing MFCC parameters for accurate real-time classification of breathing phases. Furthermore, we benchmark the optimized MFCC configuration against multiple state-of-the-art deep learning models (VGGish, YAMNet, MobileNetV2) to assess the robustness and generalizability of the proposed settings. By doing so, we demonstrate that an appropriately optimized classical machine learning pipeline can achieve performance comparable to deep learning models while offering greater interpretability and lower computational cost—an important consideration for clinical and mobile health applications.

This paper is organized as follows. Section 2 reviews related work on breathing phase detection and MFCC applications in audio signal processing, providing a foundation for the proposed approach. Section 3 details the materials and methods, including dataset description, MFCC parameter configurations, and the machine learning framework employed. Section 4 presents experimental results, evaluating the performance of optimized MFCC parameters in detecting breathing phases. Section 5 discusses the findings, their implications, limitations, and future research directions. Section 6 concludes the paper by summarizing the key contributions.

## 2. Related Work

The concept of MFCC was introduced by Davis and Mermelstein in the late 20th century as a compact and robust feature extraction method for automatic speech recognition. MFCC combines the mel scale, designed to approximate human auditory perception, with cepstral coefficients to represent the short-term power spectrum of audio signals [23]. In biomedical applications, particularly in respiratory sound analysis, MFCC gained attention starting from the mid-2000s. They have been widely used to extract features from lung and tracheal sounds for detecting adventitious events such as wheezes, crackles, and rhonchi, as well as for classifying respiratory pathologies [26,27]. Since the 2010s, MFCC has been integrated with machine learning and deep learning frameworks to enhance accuracy in tasks like breathing phase detection and disease diagnosis from audio recordings, addressing challenges such as noise robustness and signal variability [28,29,30].

Various approaches have been proposed to optimize MFCC parameters for enhanced performance in respiratory sound analysis. Bardou et al. [31] employed convolutional neural networks for lung sound classification, integrating MFCC-like features to achieve high accuracy in distinguishing normal and abnormal respiratory events, highlighting synergy with deep learning in medical diagnostics. Dash et al. [32] proposed a bio-inspired cepstral method to detect COVID-19 from speech. It showed adaptability in spotting respiratory impairments via pattern recognition. Ingco et al. [33,34] advanced an enhanced MFCC framework for respiratory sound analysis, optimizing parameters to capture breathing frequency characteristics and evaluating an intelligent classifier that improved pathological sound detection with machine learning in noisy environments.

In the context of breathing phases, audio-based methods employing MFCC have been adapted for classifying pause, inhalation, and exhalation periods but often rely on default reference features [8,35,36,37]. While research by Tirronen et al. [38] explored the role of frame length in identifying voice pathologies, it did not extend to evaluating hop length or the quantity of coefficients. In a related advancement, Yan et al. [25] investigated the optimization of MFCC parameters for the automated identification of respiratory diseases, achieving notable enhancements in classification accuracy through adjustments to frame length, hop length, and coefficient count. However, their work focused on pathology detection rather than explicit breathing phase classification.

## 3. Materials and Methods

### 3.1. Dataset Description

A proprietary respiratory sound dataset was collected specifically for this study, comprising 1500 manually annotated audio segments evenly distributed among three classes: inhalation, exhalation, and pause (500 samples per class). Recordings were obtained from five healthy adult participants (three males, two females; age range: 27–35 years) in a controlled indoor environment to minimize external noise interference. Audio was captured using two consumer-grade devices: the built-in microphone of a laptop and a mobile device, positioned on a table opposite the participants at a distance of approximately 20 cm from the mouth to ensure consistent signal capture. Participants were instructed to maintain a seated position in a relaxed posture facing the table, promoting uniformity and reducing movement artifacts during recording sessions. All recordings were saved as uncompressed WAV files (mono, 16-bit PCM, 44.1 kHz sampling rate), trimmed, and manually annotated using Audacity software (version 3.7.4) to ensure consistent quality and facilitate subsequent analysis.

Annotations were performed based on acoustic waveforms and spectrograms, delineating phases by amplitude thresholds. The annotation process entailed two independent researchers classifying breathing phases as “inhalation” and “exhalation” using acoustic waveform and spectrogram analysis with amplitude-based phase boundaries. A third researcher evaluated these labels, resolving discrepancies via consensus to maintain precision across all segments. A “pause” category was added for unclassified intervals, representing airflow cessation critical to the natural breathing cycle and marked by low acoustic activity [39], thereby improving the depiction of the full respiratory process.

**Figure 1:**
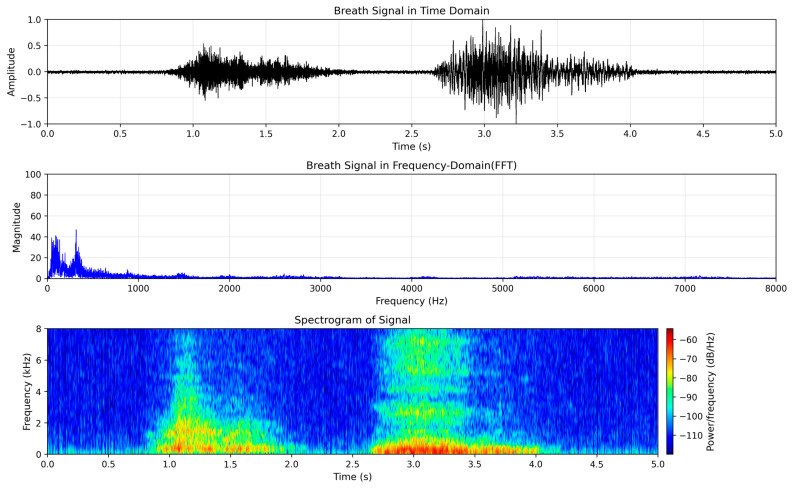
Representative analysis of normal breath sounds: (**upper**) time-domain waveform; (**middle**) frequency spectrum (FFT); (**lower**) spectrogram.

Normal breath sounds from the dataset, as illustrated in Figure 1, consist of two primary phases—inhalation and exhalation—separated by short pauses. The time-domain waveform displays distinct amplitude bursts corresponding to these phases, while the frequency-domain analysis (FFT) shows a dominant peak at 300 Hz, with additional components at approximately 50 Hz and 100 Hz. In the spectrogram, both phases are clearly distinguishable, with energy concentrations around 90 Hz, 400 Hz, and 1600 Hz persisting throughout the signal, which reflects typical spectral characteristics of healthy respiratory patterns.

### 3.2. Pre-Processing

Prior to feature extraction, all audio recordings were resampled to 16 kHz, converted to mono, and normalized to zero mean and unit variance. Segments shorter than the designated frame length were zero-padded to ensure uniform input dimensions. Labels were encoded using one-hot or integer encoding as required for specific classification models. No additional filtering or denoising was applied in this controlled study to preserve the original signal characteristics and evaluate baseline performance. For field deployment, where environmental noise from ambient sources, such as traffic or conversations, may contaminate respiratory sound recordings, implementing denoising strategies is essential to enhance signal quality and model robustness.

### 3.3. Mel Frequency Cepstral Coefficients Feature Extraction

MFCCs are a standard feature set for audio signal analysis, widely used in speech processing and biomedical applications due to their effectiveness in capturing perceptually relevant spectral characteristics of sound.

The extraction process consists of several stages: segmentation of the raw audio signal into short, overlapping frames; application of a windowing function (Hamming window) to reduce spectral leakage; transformation into the frequency domain using the FFT; mapping of the power spectrum onto the mel scale via a filterbank; logarithmic compression; and finally, decorrelation and dimensionality reduction via the discrete cosine transform [24,40,41].

The MFCCs for each frame are computed as shown in Equation (1):

(1) $$C_{m}=\sum_{j=0}^{J-1}\cos\left(m\frac{\pi }{J}(j+0.5)\right)\log_{10}\left(E_{j}\right),\;\;\;\;\;0\leq m\leq L-1$$

where $E_{j}$ is the energy of the j-th mel filterbank, *J* is the total number of mel bands, *L* is the number of cepstral coefficients, and m is the index of the coefficient [25].

**Figure 2:**
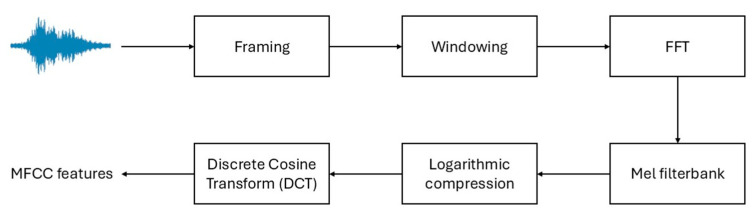
Schematic overview of the MFCC extraction pipeline.

MFCCs were selected as the primary feature representation due to their proven effectiveness in respiratory and biomedical audio analysis [25]. Feature extraction followed a standard pipeline as shown in Figure 2.

A stepwise parameter optimization strategy was employed. First, the number of coefficients varied within the range 13–80 while other parameters were kept at default values. The optimal value was fixed and used as a baseline for the subsequent tuning of frame length (25–800 ms) and finally hop length (5–500 ms). The MFCC feature vectors were obtained by calculating the mean of the frame-wise MFCC coefficients across the entire segment. The extraction of MFCC features from each audio recording was performed using the Librosa library [42].

### 3.4. SVM Classifier

A Support Vector Machine (SVM) classifier was implemented using the Scikit-learn library to evaluate the discriminative power of the optimized MFCC features [43]. SVM is a widely used and versatile algorithm known for its ability to construct optimal separating hyperplanes in high-dimensional feature spaces [44]. The RBF kernel was employed to allow for nonlinear separation in the feature space. The regularization parameter C=1.0 was chosen to balance training error minimization with margin maximization, while the gamma parameter was set to “scale”, automatically adapting to the variance of the input features and controlling the influence of individual support vectors. MFCC feature vectors, computed as the mean of frame-level coefficients, were standardized to zero mean and unit variance within each fold.

Model performance was assessed using 10-fold stratified cross-validation, implemented via the StratifiedKFold function from the Scikit-learn library, with a fixed random seed of 42 to ensure reproducibility [43]. This approach divided the dataset into ten folds, with nine folds used for training and one-fold used for testing in each iteration. The process was repeated ten times, ensuring that each fold served as the test set exactly once. Stratification preserved the proportion of classes across all folds, thereby preventing potential class imbalance bias. The results from all folds were aggregated, and mean values with standard deviations were reported for accuracy, AUC, F1-score, precision, and equal error rate (EER).

## 4. Results

We systematically investigated the influence of three critical MFCC extraction parameters—number of coefficients, frame length, and hop length—on the accuracy of respiratory phase classification using an SVM classifier. In addition to traditional feature-based approaches, we benchmarked the optimized MFCC configuration against a range of deep learning and pretrained audio embedding methods, including VGGish, YAMNet, and MobileNetV2, to comprehensively assess model performance on the same dataset.

### 4.1. Number of Coefficients

The number of MFCC coefficients determines the amount of spectral information retained from the original signal, with higher values theoretically capturing more detail but potentially introducing redundancy. In this study, we systematically varied the number of coefficients from 13 to 80, where 13 corresponds to the minimal widely adopted MFCC set and 80 represents an extended, information-rich configuration. The frame length and hop length were both fixed at default values (25 ms and 10 ms, respectively) to isolate the effect of this parameter.

**Figure 3:**
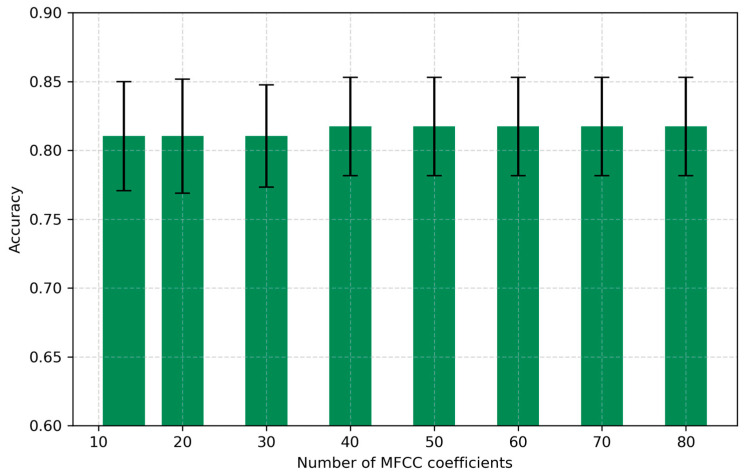
Effect of the number of MFCC coefficients on accuracy.

As shown in Figure 3, the classification accuracy remained stable for n_mfcc between 13 and 30 (accuracy ≈ 0.8104 ± 0.0396–0.0371), with a slight improvement observed at 40 and higher values (accuracy up to 0.8173 ± 0.0358 for n_mfcc = 50 and above). However, the relative gain in accuracy beyond 30 coefficients was marginal, suggesting that the optimal trade-off between informative content and computational complexity is achieved at or slightly above 30 coefficients. Therefore, n_mfcc = 30 was selected as the optimized setting for subsequent experiments.

### 4.2. Frame Length

The extraction of MFCC features begins by segmenting the original time-domain signal into short frames, where the duration of each frame—termed the frame length—plays a critical role in capturing the temporal dynamics of the signal. This step is essential because the spectral content of respiratory sounds evolves over time, and a Fourier transform performed over the entire recording would obscure these changes.

**Figure 4:**
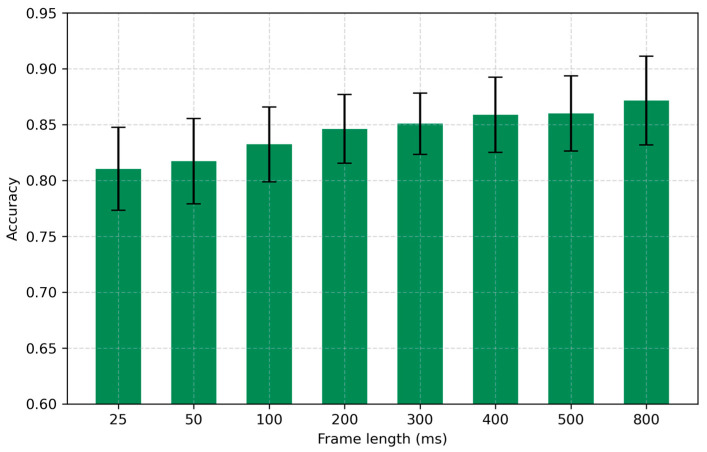
Effect of frame length on accuracy.

In this study, we systematically varied the frame length from 25 ms to 800 ms, while keeping the number of MFCC coefficients and the hop length fixed at their optimized or default values (n_mfcc = 30, hop length = 10 ms). As presented in Figure 4, the classification accuracy increased steadily with longer frame lengths: from 0.8104 ± 0.0371 at 25 ms up to 0.8716 ± 0.0397 at 800 ms. Notably, the largest gains were observed when increasing the frame length above 100 ms, suggesting that longer windows allow for the aggregation of more robust time-frequency information relevant to respiratory phase classification. Based on these results, a frame length of 800 ms was selected as optimal for subsequent experiments.

### 4.3. Latency-Accuracy Trade-Off

Increasing the frame length improves classification accuracy but also introduces proportional latency due to the longer analysis windows, which is critical for real-time respiratory monitoring (Table 1). In respiratory sound analysis, frame lengths of 200–300 ms are often considered optimal, offering a balanced compromise between responsiveness and analytical robustness [9]. A 300 ms window was therefore selected as a practical configuration, ensuring high accuracy with acceptable delay. Additionally, hop length impacts analysis speed: smaller hop values lead to denser frame overlaps and higher computational load per second of audio.

### 4.4. Hop Length

The hop length parameter specifies the number of samples shifted between consecutive frames during MFCC extraction. Shorter hop lengths result in greater frame overlap, which reduces spectral leakage and allows for a more continuous and robust representation of the signal. In this study, hop length was varied from 5 ms to 500 ms, with n_mfcc fixed at 30.

**Figure 5:**
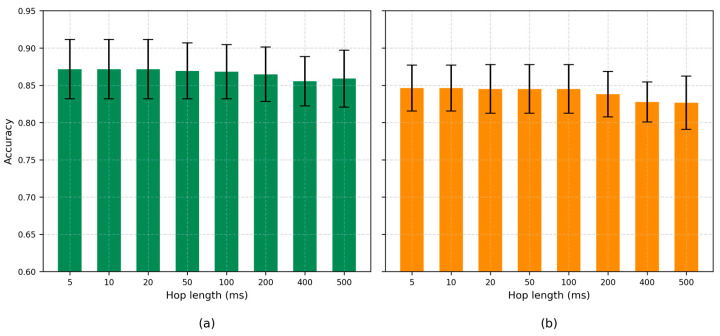
Effect of hop length on classification accuracy: (**a**) Optimized frame length of 800 ms; (**b**) Real-time motivated frame length of 300 ms.

The influence of hop length on classification accuracy was assessed for both the optimized frame length (800 ms) and a clinically motivated setting (300 ms). As illustrated in Figure 5, shorter hop lengths (5–20 ms) consistently produced the highest and most stable accuracy, regardless of window duration. Accuracy declined gradually as hop length increased beyond 100 ms, with more pronounced decreases observed for hop lengths above 200 ms. These results confirm that substantial frame overlap is beneficial for robust respiratory phase classification across both optimal and low-latency scenarios. A hop length of 10 ms was therefore selected for all further experiments.

### 4.5. Optimal Combination of Parameters

A systematic grid search across MFCC extraction parameters was performed using nested loops, evaluating every possible combination. This exhaustive evaluation revealed that the combination of 30 coefficients, an 800 ms frame length, and a 10 ms hop length yields optimal performance for respiratory phase classification using an SVM classifier. This configuration resulted in the highest accuracy (0.8716 ± 0.0397) and consistent improvements across all other metrics, including AUC, F1-score, precision, and EER, as summarized in Table 2. The default configuration employs the standard settings commonly used in audio processing literature: 13 MFCC coefficients (the minimal widely adopted set), a frame length of 25 ms, and a hop length of 10 ms. In contrast, the worst configuration represents the poorest-performing combination encountered in the parameter sweeps, consisting of 13 MFCC coefficients, a frame length of 25 ms, and a hop length of 500 ms.

**Figure 6:**
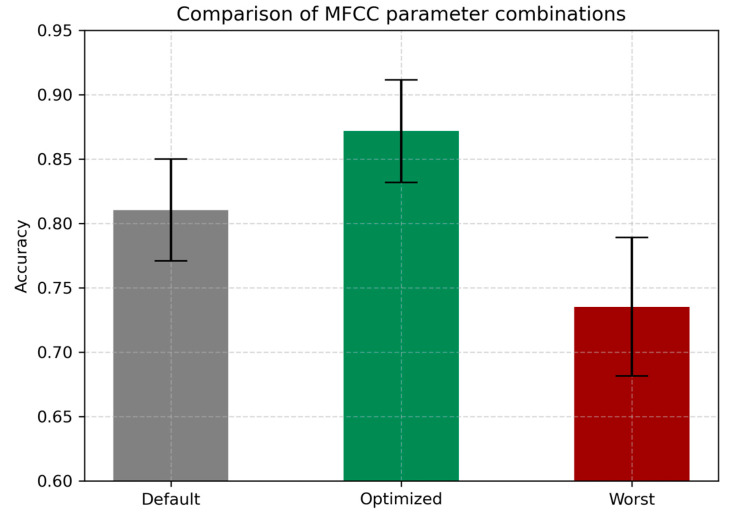
Accuracy of SVM classifier for MFCC parameter configurations.

The advantage of parameter optimization is also depicted in Figure 6, which demonstrates a marked increase in classification accuracy for the optimized configuration compared to both the default and worst-case settings. These findings underscore the importance of data-driven MFCC tuning for automatic analysis of respiratory sounds.

### 4.6. Comparison with Deep Learning Models

Pretrained VGGish [45] and YAMNet [46] models, recognized as state-of-the-art audio feature extractors, were used to generate fixed-length embeddings (128 dimensions for VGGish and 1024 for YAMNet). VGGish is a 24-layer convolutional neural network built on the VGGNet architecture [47], while YAMNet employs a MobileNet-v1 backbone and is optimized for audio event classification in the AudioSet corpus [48]. Both models were used in frozen mode without fine-tuning, and the averaged embeddings were classified using a Random Forest (RF). MobileNetV2, pretrained on ImageNet, was evaluated as a feature extractor for both MFCC and log-mel spectrogram representations [49]. The frozen backbone of MobileNetV2 produced 1280-dimensional feature vectors that were subsequently classified by an RF.

As summarized in Table 3, the SVM trained on optimized MFCC features achieved the highest overall performance. Among deep learning approaches, VGGish + RF performed best, while YAMNet + RF showed the lowest results. Although deep learning models based on pretrained embeddings are effective for general audio classification, classical models such as SVM remain advantageous due to their computational efficiency and interpretability, which are particularly important in clinical and resource-limited settings.

## 5. Discussion

This study demonstrates that systematic optimization of MFCC extraction parameters markedly enhances the accuracy of automated respiratory phase classification. The optimal parameter set (30 coefficients, 800 ms frame length, 10 ms hop length) consistently outperformed default and suboptimal settings across all evaluation metrics. When compared to deep learning and embedding-based models (VGGish, YAMNet, MobileNetV2), the optimized SVM model achieved equivalent or superior results with significantly lower computational requirements and greater interpretability. This underscores the ongoing relevance of classical machine learning for biomedical audio tasks, particularly where data is limited and transparency is essential. A key translational insight is the trade-off between accuracy and latency: longer frame lengths maximize accuracy but increase response time, potentially limiting real-time clinical usability. Our results indicate that 200–300 ms frames preserve clinically meaningful accuracy (≥0.8462) while keeping latency well below accepted thresholds in respiratory monitoring applications.

Compared to prior research, our findings emphasize the critical role of MFCC parameter tuning for breathing phase detection. Previous studies summarized in Table 4 primarily focused on other problems and modalities. For example, Tirronen et al. [38] applied default MFCC settings for voice pathology detection using speech data from the SVD dataset, varying only the frame length to assess its impact, while keeping the number of coefficients and hop length fixed. In contrast, Yan et al. [25] and our study systematically explored all three key MFCC parameters across multiple datasets and tasks. Importantly, unlike speech or cough, breath sounds exhibit longer, lower-frequency structures and slower temporal dynamics, particularly in resting or paced breathing.

The current study is subject to several limitations that warrant careful consideration for interpreting the results and guiding future investigations. The proprietary dataset includes recordings of five healthy adult participants in a controlled indoor environment, limiting generalizability. Moreover, the dataset lacks diversity in pathological conditions (e.g., asthma, pneumonia, COPD), demographics (e.g., pediatric or elderly populations), and recording devices. Although both a laptop and a smartphone were used, broader variation across device types was not explored. The absence of environmental noise, such as background sounds typical in real-world settings, may overestimate the robustness of the optimized MFCC parameters in practical applications. Although no denoising strategies were employed in the present study, their implementation is crucial for field deployment, where ambient noise from sources like traffic or conversations could compromise respiratory sound recordings, thereby improving signal integrity and model resilience. Traditional approaches, including spectral subtraction and Wiener filtering, are well-known but often involve a trade-off between noise suppression and the preservation of diagnostically relevant high-frequency components, such as subtle crackles or wheezes [50,51,52,53]. Similarly, Butterworth band-pass filtering, while simple and computationally efficient, imposes strict frequency cutoffs (typically 100–2000 Hz) and risks attenuating clinically meaningful information above this range [54,55]. Wavelet-based denoising, despite its adaptability to non-stationary signals, can lead to partial loss of transient features due to its reliance on fixed basis functions [56,57]. More advanced strategies, such as adaptive filtering, have shown promise in preserving the acoustic signatures while reducing background noise overhead [58]. However, these methods require careful tuning and validation to avoid over-smoothing or artifact introduction. Future studies should explore the integration of hybrid or machine-learning-based denoising pipelines, tailored to the spectral and temporal characteristics of respiratory sounds, to ensure robust performance in real-world noisy environments. These omissions may reduce model performance in noisy environments or heterogeneous patient cohorts, where atypical breathing patterns and signal artifacts pose additional challenges.

To address these limitations, future work should prioritize external validation using multi-center studies and publicly available respiratory sound datasets with broader variability. The ICBHI 2017 [59] database includes recordings from patients with various pulmonary pathologies, Coswara [60] offers noisy and ambulatory samples from individuals with COVID-19, and DeepBreath [61] provides pediatric data collected across multiple sites. These resources enable testing across clinical, demographic, and acoustic conditions. In addition, combining audio with physiological signals and applying denoising techniques (e.g., spectral subtraction or bandpass filtering) may further improve robustness in real-world deployments.

## 6. Conclusions

This study advances the understanding of respiratory sound analysis by demonstrating that MFCC parameter optimization can significantly improve phase detection while retaining model interpretability. Our findings challenge the common reliance on deep learning by showing that classical models, when combined with optimized features, remain competitive. Beyond technical performance, these insights have practical implications for developing lightweight, real-time respiratory monitoring solutions applicable in mobile health, telemedicine, and digital therapeutics. Future work should extend these findings to larger datasets, noisy real-world environments, and hybrid feature-learning frameworks.

## Figures and Tables

**Table 1:** Trade-off between MFCC frame length, system latency, and classification accuracy.

Frame Length (ms)	Latency (s)	Accuracy (±STD)
200	0.200	0.8462 ± 0.0308
300	0.300	0.8508 ± 0.0273
400	0.400	0.8589 ± 0.0337
800	0.800	0.8716 ± 0.0397

**Table 2:** Comparison of SVM model performance.

Configuration	Accuracy	AUC	F1	Precision	EER
Worst	0.7352	0.8855	0.7437	0.7382	0.1760
Default	0.8096	0.9338	0.8165	0.8079	0.1295
Optimized	0.8716	0.9663	0.8764	0.8725	0.0899

**Table 3:** Comparison of optimized SVM and deep learning-based approaches.

Model/Feature	Accuracy	AUC	F1	Precision	EER
SVM (MFCC, optimized)	0.8716	0.9663	0.8764	0.8725	0.0899
VGGish + RF	0.8713	0.9598	0.8802	0.8822	0.0960
YAMNet + RF	0.7871	0.9372	0.7984	0.7952	0.1295
MobileNetV2 + MFCC	0.8416	0.9594	0.8548	0.8538	0.1088
MobileNetV2 + Spectrogram	0.8416	0.9661	0.8496	0.8517	0.1041

**Table 4:** Previous work on MFCC parameter optimization for respiratory sound analysis.

Study	Problem	Dataset	Modality	Classifier	n_mfcc	Frame Length (ms)	Hop Length (ms)	Accuracy
Tirronen et al., 2024 [38]	Voice pathology detection	SVD ^1^	Speech	SVM	13 (default)	500	5 (default)	66.4%
Yan et al., 2025 [25]	Automatic detection of respiratory diseases	Cambridge COVID-19 Sound database	Speech, Cough, and Breath	SVM/LSTM	30	25	5	81.1%/79.2%
		Coswara	Speech, Cough, and Breath	SVM/LSTM	40	25	5	80.6%/79.6%
		SVD ^1^	Speech	SVM/LSTM	30	25	5	71.7%/71.9%
Current study	Breathing phase detection	Proprietary dataset	Breath	SVM	30	300	10	85.08%

^1^ Saarbrücken voice disorders.

## Data Availability

The datasets for this article are not publicly available due to concerns regarding participant anonymity. Requests to access the dataset should be directed to the corresponding author.

## Abbreviations

The following abbreviations are used in this manuscript:

AUC	Area Under the Curve
COPD	Chronic Obstructive Pulmonary Disease
COVID-19	Coronavirus Disease 2019
EER	Equal Error Rate
FFT	Fast Fourier Transform
LSTM	Long Short-Term Memory
MFCCs	Mel Frequency Cepstral Coefficients
OSA	Obstructive Sleep Apnea
PCM	Pulse Code Modulation
RBF	Radial Basis Function
RF	Random Forest
ROC	Receiver Operating Characteristic
STD	Standard Deviation
SVD	Saarbrücken Voice Disorders
SVM	Support Vector Machine
WAV	Waveform audio file format
